# Convolutional neural networks reveal differences in action units of facial expressions between face image databases developed in different countries

**DOI:** 10.3389/fpsyg.2022.988302

**Published:** 2022-11-03

**Authors:** Mikio Inagaki, Tatsuro Ito, Takashi Shinozaki, Ichiro Fujita

**Affiliations:** ^1^Graduate School of Frontier Biosciences, Osaka University, Suita, Osaka, Japan; ^2^Center for Information and Neural Networks, National Institute of Information and Communications Technology Suita, Osaka, Japan; ^3^School of Engineering Science, Osaka University, Toyonaka, Osaka, Japan; ^4^Faculty of Informatics, Kindai University, Higashi-Osaka, Osaka, Japan; ^5^Research Organization of Science and Technology, Ritsumeikan University, Kusatsu, Shiga, Japan

**Keywords:** facial expression, emotion, facial movement, transfer learning, supervised learning, cultural universality, AlexNet, action unit

## Abstract

Cultural similarities and differences in facial expressions have been a controversial issue in the field of facial communications. A key step in addressing the debate regarding the cultural dependency of emotional expression (and perception) is to characterize the visual features of specific facial expressions in individual cultures. Here we developed an image analysis framework for this purpose using convolutional neural networks (CNNs) that through training learned visual features critical for classification. We analyzed photographs of facial expressions derived from two databases, each developed in a different country (Sweden and Japan), in which corresponding emotion labels were available. While the CNNs reached high rates of correct results that were far above chance after training with each database, they showed many misclassifications when they analyzed faces from the database that was not used for training. These results suggest that facial features useful for classifying facial expressions differed between the databases. The selectivity of computational units in the CNNs to action units (AUs) of the face varied across the facial expressions. Importantly, the AU selectivity often differed drastically between the CNNs trained with the different databases. Similarity and dissimilarity of these tuning profiles partly explained the pattern of misclassifications, suggesting that the AUs are important for characterizing the facial features and differ between the two countries. The AU tuning profiles, especially those reduced by principal component analysis, are compact summaries useful for comparisons across different databases, and thus might advance our understanding of universality vs. specificity of facial expressions across cultures.

## Introduction

Culture-related similarities and differences in facial expression communications have been the subject of intense debate over the past few decades (Ekman et al., 1992; Izard, 1994; Russell, 1994; Jack, 2013; Barrett et al., 2019). On the one hand, facial expressions are suggested to be universal across cultures. Ekman and colleagues showed that a set of photographs of facial expressions of six basic emotions (happy, fearful, sad, angry, surprised, and disgusted) resulted in mostly the same recognition of emotions across different cultures (Ekman et al., 1969). On the other hand, cultural differences in recognizing facial expressions have often been demonstrated. For instance, facial expressions displayed by individuals in a cultural group are more accurately recognized by observers of the same group than those of other cultural groups (Elfenbein and Ambady, 2002). Recognition of facial expressions relies on culture-specific facial features in addition to those shared by different cultures.

A key step to understanding the culture-specific and -nonspecific aspects of facial expression recognition is to clarify whether and how a specific emotion is consistently associated with specific patterns of facial movements across different cultures. This requires quantifying the similarities and dissimilarities of the facial movement patterns in different cultures, and should rely on large-scale data to identify the cultural variability beyond simply the inter-individual fluctuations that occur within a single culture. Facial movement patterns can be quantitatively characterized with a framework of action units (AUs) defined in the facial action coding system (FACS; Ekman and Friesen, 1976; Ekman et al., 2002). Each AU represents a specific facial movement generated by particular facial muscles. The combinations of AUs comprehensively describe the entire patterns of facial movements displayed by humans.

In this study we applied the framework of AU to characterize the visual features of facial expressions in two image databases of facial expressions created in different countries: the Karolinska Directed Emotional Faces (KDEF) database, developed in Sweden (Lundqvist et al., 1998), and the Kokoro Research Center (KRC) facial expression database, developed in Japan (Ueda et al., 2019). The two databases consist of facial photographs of several tens of posers, each displaying the six basic expressions and neutral one, taken from several different directions. Because the posers of each database live in geographically distant and culturally distinct countries, their facial behaviors most likely reflect cultural differences between the two countries.

We took an approach of using convolutional neural networks (CNNs) to analyze these image databases. A CNN, consisting of several computational layers serially connected by variable weights, undergoes supervised learning and thereby learns to classify each input into one output category (LeCun et al., 2015). In the case of image classification, a CNN adapts itself to the image database used for training and thus acquires database-specific feature representations that are useful for the classification. CNNs are constructed to share a similar architecture with the visual system of the brain (Hassabis et al., 2017). Feature representation in the CNNs constructed this way has been shown to be similar to that of the brain (Yamins et al., 2014; Güçlü and van Gerven, 2015; Yamins and DiCarlo, 2016). Importantly, a subset of feature representations selective for AUs is suggested to be useful for classifying facial expressions (Zhou and Shi, 2017). These previous findings motivated us to use CNNs to quantify selectivity to AUs.

We show that CNNs learned to classify facial expressions of the basic emotions (and neutral faces) in database-specific manners. The ability of the CNNs for classifying the facial expressions was not fully generalized between the KDEF and KRC databases, suggesting that the visual features of the facial expressions differ between Sweden and Japan. Analysis of the final layers of the CNNs revealed that their outputs were selective to subsets of the AUs. The patterns of this AU selectivity differed considerably between the two databases. Similarities and dissimilarities of the AU selectivity between the databases might reflect cultural commonalities and differences in the encoding of facial expressions.

## Materials and methods

### Image database

**Figure 1:**
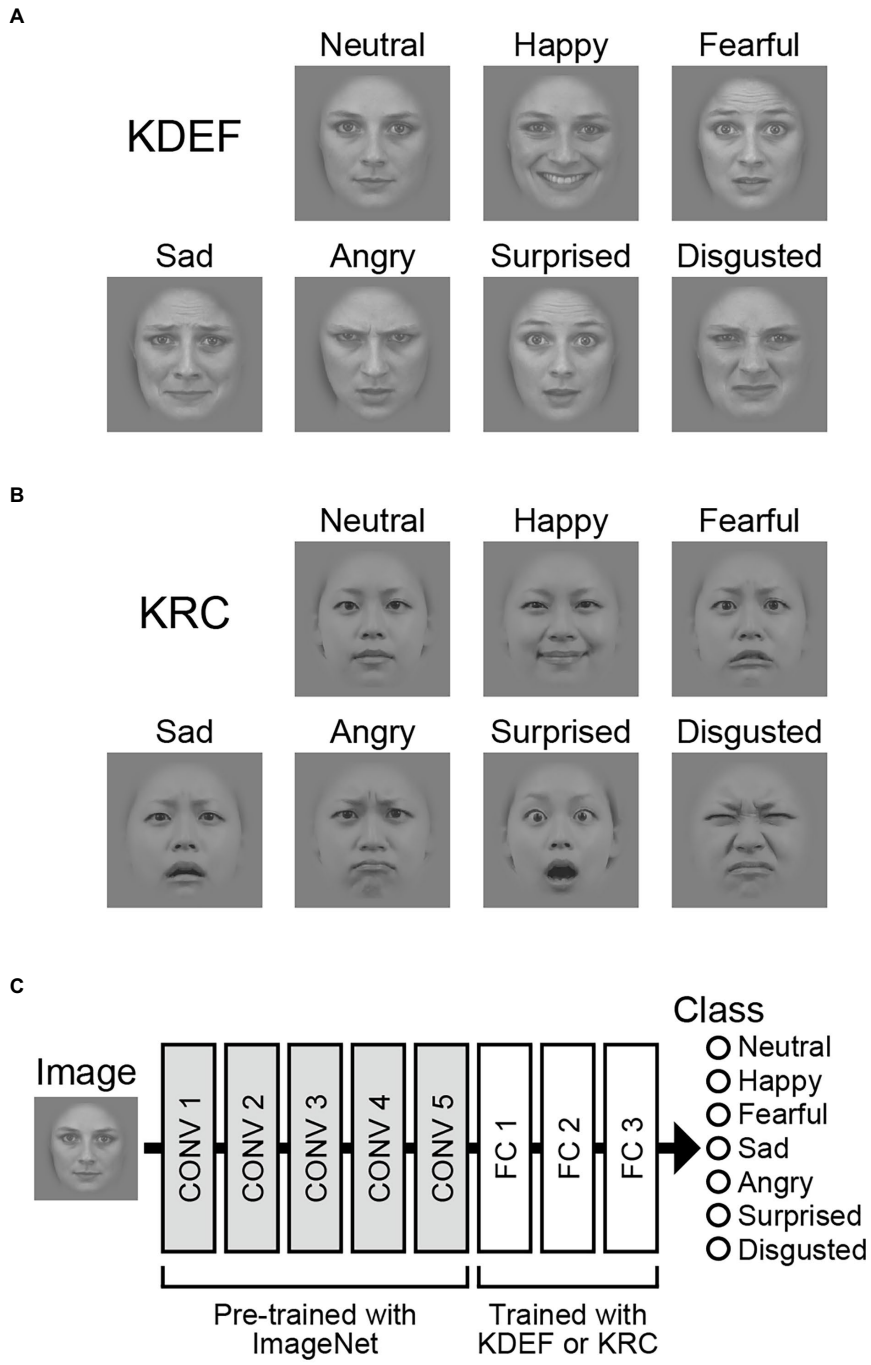
Input images and the architecture of the CNN. Example images of seven facial expressions in the KDEF database **(A)** and those in the KRC database **(B)**. Examples are BF01NES, BF01HAS, BF01AFS, BF01SAS, BF01ANS, BF01SUS, and BF01DIS from the KDEF database, and fd19_neu_d, fd19_hap_d, fd19_fea_d, fd19_sad_d, fd19_ang_d, fd19_sur_d, and fd19_dis_d from the KRC database. The CNN consisted of five convolutional (CONV) layers and three fully connected (FC) layers **(C)**. The weights of the convolutional layers were adopted from “AlexNet,” which was pre-trained for classification of object images in the ImageNet database (Russakovsky et al., 2015). We trained our CNNs to optimize the weights of the FC layers to classify the seven facial expressions through the experimental runs.

We used two image databases of facial expressions: the Karolinska Directed Emotional Faces (KDEF) database, developed in Sweden (Figure 1A; Lundqvist et al., 1998), and the Kokoro Research Center (KRC) facial expression database, developed in Japan (Figure 1B; Ueda et al., 2019). The KDEF database comprises faces of 70 individuals, and the KRC database faces of 74 individuals. From each database, we selected 60 individuals who were facing forward and who displayed seven different facial expressions. These were labeled as neutral, happy, afraid, sad, angry, surprised, and disgusted in the KDEF database, and neutral, happy, fearful, sad, angry, surprised, and disgusted in the KRC database. We adopted the KRC labels for both databases to simplify the terminology in this study.

Original color photographs in the KDEF and KRC databases were pre-processed by the following procedures. After converting into grayscale images, they were fed into a face detection algorithm of a computer vision library, OpenCV,^1^ to extract the facial region. Then, the pixel intensity histogram of the facial region was adjusted in each image so that the mean and standard deviation of the histogram became 128 and 32, respectively. Finally, irrelevant features outside of the facial region such as body and background scene were removed by a mask which had a smooth boundary with the center oval region.

### Network

We used a CNN that was pre-trained to classify images into 1,000 different object groups (“AlexNet”; Krizhevsky et al., 2012). The CNN consists of five convolutional layers (CONV1–5) and three fully connected layers (FC1–3; Figure 1C). Max pooling operations were implemented after the CONV1, CONV2, and CONV5 layers. We replaced the three FC layers with new ones that were subjected to training of discriminating between the seven facial expressions. The new FC1, FC2, and FC3 layers contained 4,096, 4,096, and seven units, respectively. A dropout process was added before the FC1 and FC2 layers, and the proportion of units dropped out of each weight update was set to 0.1. This procedure was taken for facilitating learning across all units. For each input image, the seven computational units in the FC3 layer computed the score of the corresponding facial expressions. The scores ranged from 0 to 1 after a softmax operation, and were interpreted as probabilities of the classified emotion for a given input image. Before starting training with the KDEF or KRC databases, the FC layers were initialized by random weights sampled from a normal distribution (He et al., 2015). The pre-trained weights outside of the FC layers were unchanged throughout the experiments.

### Training

We trained the modified CNN to classify facial expressions by supervised learning using the emotion labels of input images as teaching signals. In each run, we randomly divided the images of 60 individuals into a training dataset (40), validation dataset (8), and test dataset (12). We periodically presented the validation dataset to the network during training to check whether overfitting to the training dataset occurred (see below). After completion of the training, we evaluated the performance of the CNNs using the test dataset. We artificially increased the number of face images by manipulating their size and position, and by reflecting them left-to-right horizontally, resulting in a total of 50 variations for each image (5 sizes × 5 positions × 2 reflections). Input size was 224 pixels × 224 pixels. In each epoch, therefore, 14,000 samples (40 × 7 × 50) and 2,800 samples (8 × 7 × 50) were used for training and validation, respectively. We further randomly divided the training set into mini-batches (32 samples each, except for the last mini-batch with 16 samples) and used stochastic gradient descent as the optimizer. We referred to a weight-updating process with a single mini-batch as an iteration. Cross-entropy was computed as “loss” (a measure of the difference between an estimated value and a true value). The training comprised 12,000 iterations over 29 epochs. We initially set the learning rate to 0.0001 for all FC layers, and updated it to 0.00001 and 0.000001 at 4,000 and 8,000 iterations, respectively. We checked the correct rate and loss for the validation dataset in each epoch to monitor signs of overfitting. For each database, we repeated the experimental runs 40 times; i.e., we trained and examined 40 CNNs each for the KDEF database and for the KRC database.

### Tuning to AUs

We examined the selectivity of the seven computational units of the FC3 layer to the AUs of facial movement by analyzing their responses to AU-manipulated face images generated by FaceGen software (Singular Inversions, Toronto, Canada). We focused on the 20 AUs listed in Table 1. These AUs are associated with one or more basic emotion(s) (Ekman et al., 2002). In the FaceGen parameter settings, we increased the intensity of one of the AUs from the minimum (0.0) to the maximum (1.0) at 11 steps so that it gradually appeared in the generated images. While we were manipulating a particular AU, we shut off the other AUs and they did not appear in the images. We thus obtained 20 series of images, each with a particular modified AU, and used them to test the selectivity of the FC3 units to the 20 AUs. Note that the generated image with the minimum intensity was identical across the 20 AUs because the image did not contain any AU (hereafter we refer to this image as the “null image”). We performed these manipulations on a face image to which we had applied the texture of the neutral female face averaged across the KDEF database available in the Averaged KDEF database (Lundqvist and Litton, 1998).

**Table 1:** List of action units (AUs) analyzed in this study.

Action unit number	Name of facial movement*
AU#1	Inner brow raiser
AU#2	Outer brow raiser
AU#4	Brow lowerer
AU#5	Upper lid raiser
AU#6	Cheek raiser
AU#7	Lid tightener
AU#9	Nose wrinkler
AU#10	Upper lip raiser
AU#11	Nasolabial furrow deepener
AU#12	Lip corner puller
AU#15	Lip corner depressor
AU#16	Lower lip depressor
AU#17	Chin raiser
AU#20	Lip stretcher
AU#22	Lip funneler
AU#23	Lip tightener
AU#24	Lip pressor
AU#25	Lips part
AU#26	Jaw drop
AU#27	Mouth stretch

* After Ekman et al. (2002).

We independently analyzed the responses (before the softmax operation) to the generated images in each computational unit. We adjusted the baseline activity by subtracting the response to the null image from the data so that the response magnitude to the null image became 0 in every computational unit.

### Correlation of response profiles

For a given pair of FC3 units, we evaluated the similarity of the selectivity by computing Spearman’s rank correlation coefficient (*rs*) between the full profiles of their responses to the AUs. We excluded the responses to the null image from the computation of the correlation coefficient because these responses were identical across the AUs, units, and CNNs as a result of our normalization procedure. The combination of the two sets of the seven units from the KDEF-and KRC-trained CNNs yielded a 7 × 7 matrix of correlation coefficients.

### Principal component analysis

We applied principal component analysis to gain insight into how AU selectivity differed across the seven facial expressions and the two databases. In most cases the shapes of the tunings were simple (e.g., monotonically increased or decreased, or having a single peak or trough); thus, we reduced the dimensionality of a response profile from 220 (20 AUs × 11 levels) to 20 by adopting a single peak or trough value for each AU. We then aligned these values taken from the seven profiles of the two CNNs to create a feature matrix (20 × 14) to which we applied principal component analysis.

We used a machine learning library, PyTorch, to perform these experiments with a GeForce 1,070 (NVIDIA, Santa Clara, CA, United States). Data were analyzed and visualized by MATLAB (Mathworks, Natick, MA, United States).

## Results

### Classification performance

**Figure 2:**
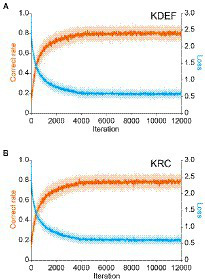
Changes in the correct rate and the loss (estimation error) during training. The correct rates (orange lines) and loss values (cyan lines) are plotted as a function of the iteration number for the KDEF-trained CNNs **(A)** and the KRC-trained CNNs **(B)**. They were computed with the training dataset. The solid lines and shaded areas represent the means and standard deviations across 40 runs, respectively. Every 10th value is plotted along the horizontal axis.

Training with the KDEF or KRC database improved the classification performance of the CNNs to a similar extent. With both databases, the average correct rate across the seven facial expressions in the training dataset rose steeply from the chance level (0.14), and surpassed 0.7 after around 2,000 iterations (Figures 2A,B; orange lines). The correct rate stayed nearly unchanged after 4,000 iterations, and reached similar levels for the two databases although it was slightly higher in the KDEF than in the KRC (average of 8,001 to 12,000 iterations, 0.80 for KDEF and 0.78 for KRC; *p* < 0.0001, *t* test). During training of each CNN with its respective training set, we periodically checked the classification performance for the validation dataset as well. The correct rate for the validation dataset also reached a plateau, indicating no sign of overfitting. The loss value for the training dataset initially sharply decreased and became relatively stable after the first 4,000 iterations both for the KDEF and KRC databases (Figures 2A,B; cyan lines). Thus, the loss value showed a profile that mirrored that of the correct rate. These results indicate that with this number of iterations, the CNNs trained with the KDEF and KRC databases obtain a high classification performance without overfitting.

**Figure 3:**
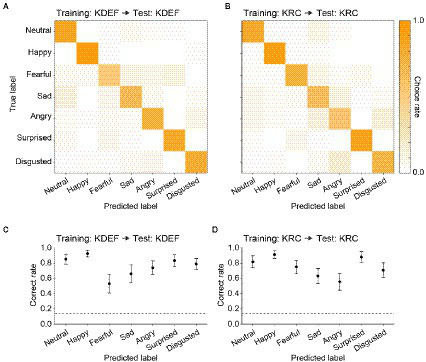
Classification performance of the CNNs in the database-matched conditions. The confusion matrices computed with the test datasets are shown for the KDEF-trained CNNs **(A)** and the KRC-trained CNNs **(B)**. The test and training datasets were derived from the same database. The mean choice rates averaged across 40 runs were coded by color (scale bar to the right). The correct rates of seven facial expressions are plotted for the KDEF-trained CNNs **(C)** and the KRC-trained CNNs **(D)**. The means and standard deviations across 40 runs are plotted. The dashed lines represent the chance level (1/7 = 0.14).

After the training, we evaluated the classification performance with the test dataset to ensure that the CNNs did not simply sort the training images into the seven facial expressions according to the instruction signals, but acquired the true ability to classify the expressions of face images including previously unseen ones. Figure 3 represents confusion matrices and correct rates of the CNNs trained with either the KDEF or KRC database. The correct rates were generally higher than the chance level (0.14; dashed lines) in both the KDEF-trained and KRC-trained CNNs. The two CNNs showed no difference in the average correct rates across the seven expressions (0.76 for KDEF-trained CNN, 0.75 for KRC-trained CNN, *p* = 0.15, *t* test). Notably, however, the way in which the correct rates depended on the facial expressions differed between the two databases (two-way ANOVA; database, *p* = 0.13; facial expression, *p* < 0.0001; interaction, *p* < 0.0001). In the KDEF-trained CNN, the correct rates were relatively high for neutral, happy, surprised, and disgusted faces, and were worst for fearful faces (Figure 3C). In the KRC-trained CNN, the correct rates were also relatively high for neutral, happy, surprised, and disgusted faces, but those for sad and angry faces were worse than those for fearful faces (Figure 3D). The high correct rates of neutral, happy, surprised, and disgusted faces suggest that facial features are consistent across exemplars of these facial expressions within each database. By contrast, fearful faces in KDEF and angry faces in KRC may be more diverse in their appearance than the other facial expressions.

**Figure 4:**
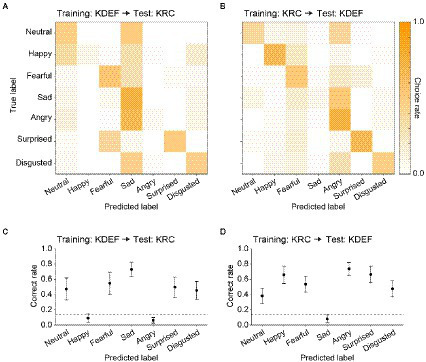
Classification performance in the database-swapped conditions. The confusion matrix **(A)** and correct rates **(C)** of the KDEF-trained CNNs when tested with the KRC database. The confusion matrix **(B)** and correct rates **(D)** of the KRC-trained CNNs tested with the KDEF database. Other conventions are the same as in Figure 3.

Given the facial expression–dependent difference in the correct rates between the KDEF-trained and KRC-trained CNNs, we next examined how well the CNNs generalized their performance to faces in the database not used for training. For this purpose, we swapped the test dataset of the used database for that of the unused database, i.e., from KDEF to KRC, or from KRC to KDEF. Confusion matrices revealed that many misclassifications of facial expressions occurred in the database-swapped conditions, while these were not found in the database-matched conditions (compare Figures 4A,B with Figures 3A,B). Higher choice rates outside of the principal diagonal in the confusion matrices were indicative of misclassifications (Figures 4A,B). For instance, the KDEF-trained CNNs often labeled neutral and angry faces as sad when they were tested with the KRC database (Figure 4A). Additionally, the KDEF-trained CNNs rarely chose happy and angry labels, resulting in much lower correct rates (near the chance level or less) for these two facial expressions (Figure 4C). Similar misclassifications also occurred when the KRC-trained CNNs were tested with the KDEF database (Figures 4B,D). In this case, the CNNs showed a clear bias against choosing the sad label for any faces (Figure 4B), and thus demonstrated poor classification performance for sad faces (Figure 4D). Overall, the correct rates in the database-swapped conditions were lower than those in the database-matched conditions in both the KDEF-trained CNNs (two-way ANOVA; matched or swapped, *p* < 0.0001; facial expression, *p* < 0.0001; interaction, *p* < 0.0001) and the KRC-trained CNNs (two-way ANOVA; matched or swapped, *p* < 0.0001; facial expression, *p* < 0.0001; interaction, *p* < 0.0001). In several cases, the correct rates even dropped to the chance level or less. As a consequence, the correct rates averaged across the seven facial expressions were lower in the database-swapped conditions than in the database-matched conditions (KDEF, 0.76 for database matched, 0.41 for database swapped, *p* < 0.0001, *t* test; KRC, 0.75 for database matched, 0.50 for database swapped, *p* < 0.0001, *t* test). Thus the ability of the CNNs to classify facial expressions was not fully generalized across the databases.

### Selectivity to AUs of facial movement

**Figure 5:**
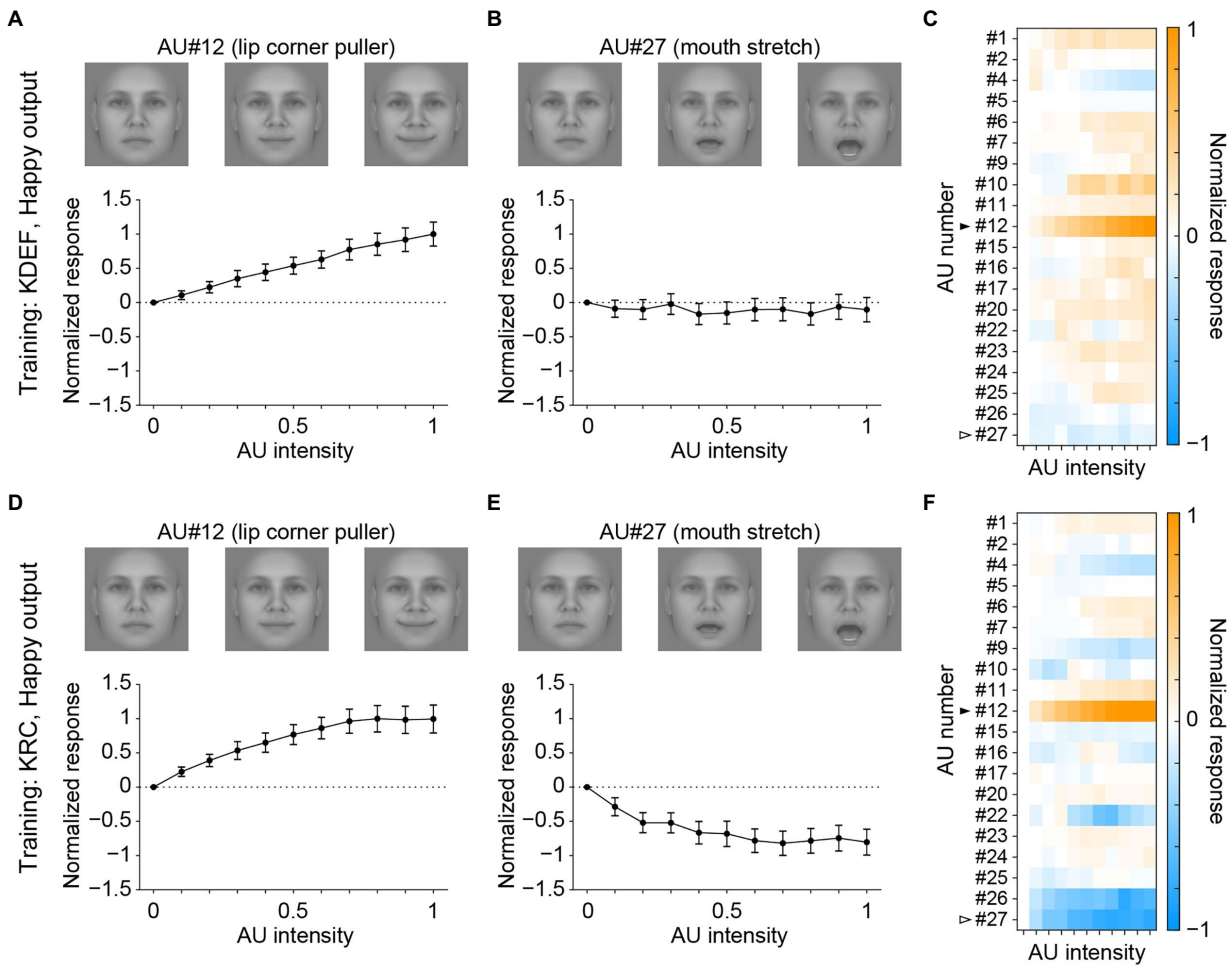
Selectivity of the outputs of the “happy” unit in the FC3 layer to the AUs. Responses of the “happy” unit of the KDEF-trained CNNs are shown for varying intensities of AU#12 (**A**, lip corner puller) and AU#27 (**B**, mouth stretch). The means and standard deviations across 40 runs are plotted. On top, three example images are shown for corresponding AU intensities of 0 (left), 0.5 (middle), and 1 (right). These images of AU manipulations are generated with the neutral female face, FNES, in the Averaged KDEF database (Lundqvist and Litton, 1998; see Tuning to AUs for details). **(C)** The response profile of the “happy” unit in the KDEF-trained CNNs to all 20 AUs with varying intensities. The mean responses averaged across 40 runs are coded by color (scale bar to the right). The responses are normalized so that the maximum absolute value of the profile becomes 1. Note that the same normalization was also applied to the data shown in **(A)** and **(B)**. The filled and open arrowheads indicate AU#12 (shown in **A**) and AU#27 (shown in **B**), respectively. **(D,E)** Tuning curves for AU#12 **(D)** and AU#27 **(E)** and the response profiles to all 20 AUs of the happy unit in the KRC-trained CNNs **(F)**. The conventions are the same as in panels **(A–C)**.

The poor classification performance in the database-swapped conditions (Figure 4) suggests that the facial pattern corresponding to each facial expression differed somewhat between the two databases. We characterized this difference by analyzing the response selectivity of the output units in the CNNs to the AUs of facial movement. We measured the responses of the seven computational units in the FC3 layer to AU-manipulated faces (see section “Tuning to AUs”). Figure 5 shows the response profiles of the “happy” units in the KDEF-trained CNNs (A–C) and the KRC-trained CNNs (D–F). Manipulation of the intensity of AU#12 (lip corner puller) elicited positive responses (relative to the response to the null image) in both the KDEF-trained CNNs (Figure 5A) and the KRC-trained CNNs (Figure 5D). Note that the other AUs were not manipulated when the effect of AU#12 was tested. The responses gradually became stronger with an increase of AU#12 intensity. These results indicate that the appearance of lip corners pulled up represented by AU#12 is a feature characterizing happy faces in both databases. Some other AUs elicited negative responses when they were set to higher intensities. For instance, AU#27 (mouth stretch) caused weak negative responses over the entire range of intensities in the KDEF-trained CNNs (Figure 5B), and gradually stronger negative responses with increased intensities in the KRC-trained CNNs (Figure 5E). Suppression of the output below the response to the null image means that the appearance of mouth stretching represented by AU#27 is indicative of non-happy faces. The full response profiles to all 20 AUs of the “happy” units were generally similar between the KDEF-trained and KRC-trained CNNs (compare Figure 5C with Figure 5F; Spearman’s correlation coefficient *rs* = 0.71, *p* < 0.0001).

**Figure 6:**
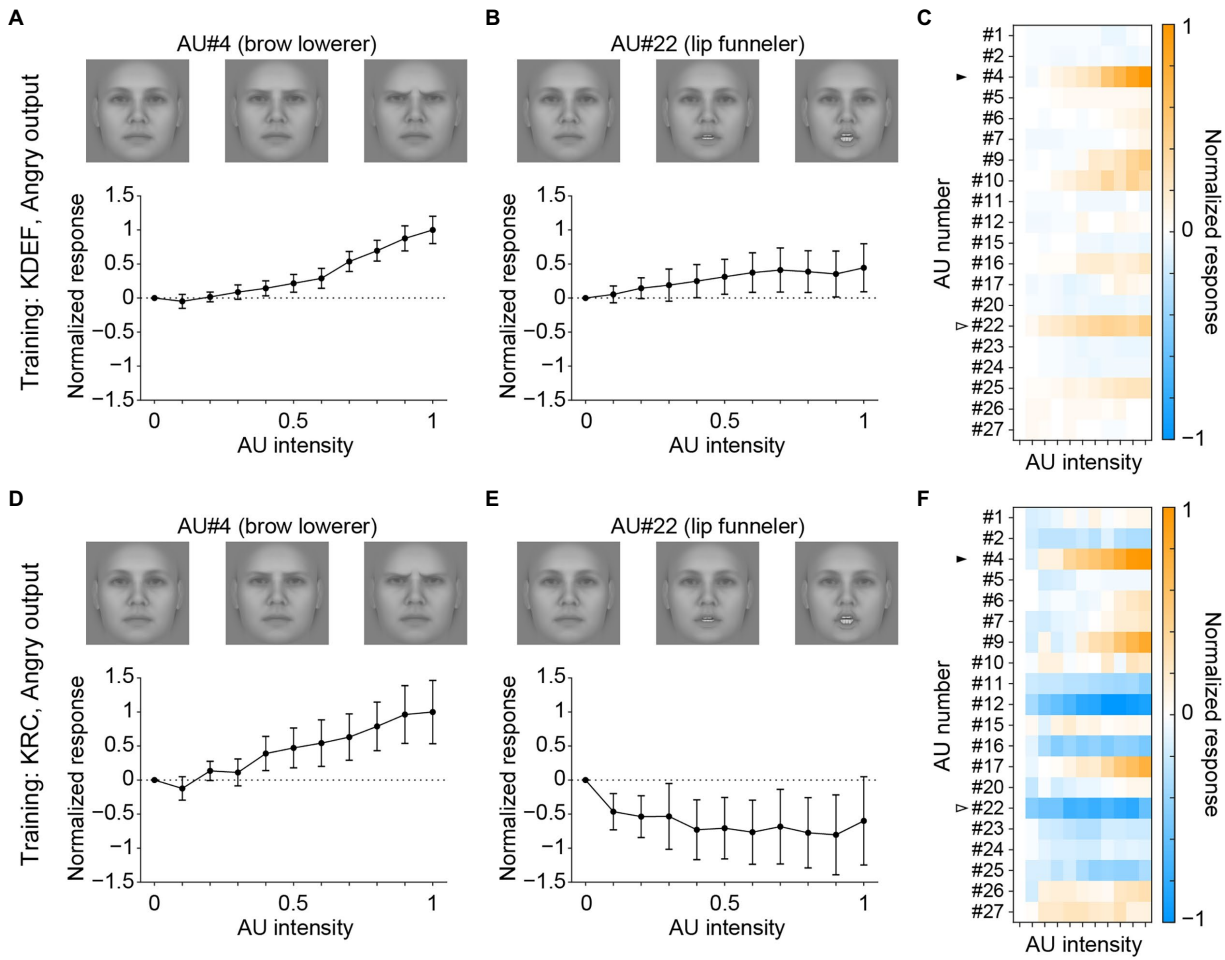
Selectivity of the outputs of the “angry” unit in the FC3 layer to the AUs. Tuning curves of the “angry” unit of the KDEF-trained CNNs for AU#4 (**A**, brow lowerer) and AU#22 (**B**, lip funneler), and the response profiles of the KDEF-trained CNNs **(C)** are shown. Lower panels **(D–F)** show the response profiles of the “angry” unit of the KRC-trained CNNs. The conventions are the same as in Figure 5.

The other facial-expression units in the FC3 layer demonstrated less similar profiles between the two groups of the CNNs. Figure 6 shows a comparison of the response profiles of the “angry” units. Higher intensities of AU#4 (brow lowerer) elicited positive responses in both the KDEF-trained (Figure 6A) and KRC-trained CNNs (Figure 6D). By contrast, AU#22 (lip funneler) had opposite effects in the two groups of CNNs: positive responses in the KDEF-trained CNNs (Figure 6B) and negative responses in the KRC-trained CNNs (Figure 6E). The lips-funneled gesture is linked with an angry face in the KDEF database, but opposes classification of the face as angry in the KRC database. The full response profiles of the “angry” units were dissimilar between the KDEF-trained and KRC-trained CNNs (Figures 6C,F; *rs* = 0.12).

**Figure 7:**
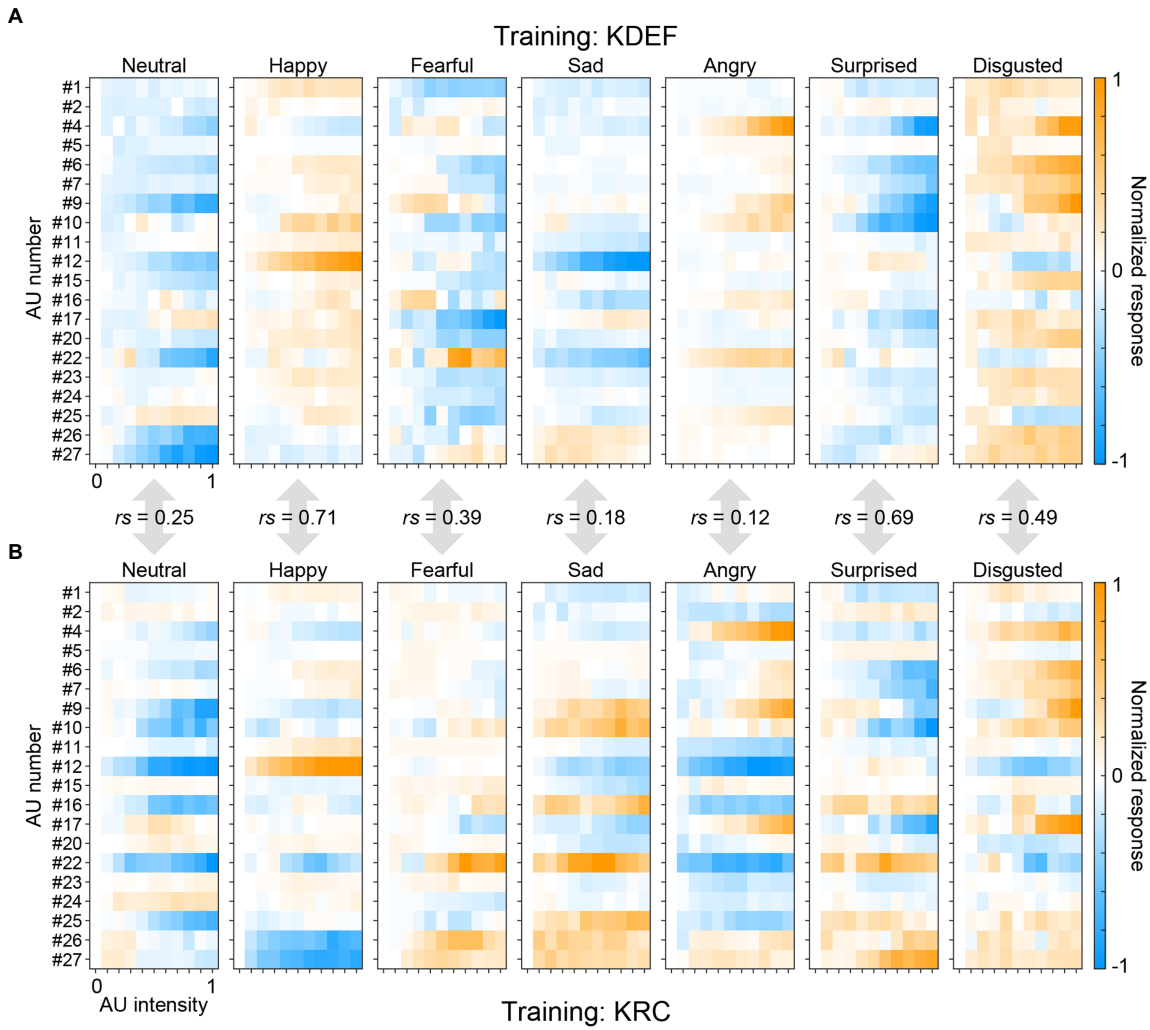
Full response profiles of the selectivity to 20 AUs. The response profiles of the seven computational units in the FC3 layer are shown for the KDEF-trained CNNs **(A)** and the KRC-trained CNNs **(B)**. The mean responses averaged across 40 runs are coded by color (scale bar to the right). In each panel, the responses are normalized so that the maximum absolute value of the entire profile becomes 1. Spearman’s rank correlation coefficients are shown for each of the seven computational units.

Figure 7 shows the full response profiles of all facial-expression units of the KDEF-trained and KRC-trained CNNs. For each facial expression, we quantified the similarity of the profiles between the two groups of the CNNs by computing Spearman’s correlation coefficients. The degree of similarity in the AU selectivity varied considerably across facial expressions. The coefficient value was highest for the “happy” units (*rs* = 0.71, *p* < 0.0001), and gradually decreased for the units in the following order: “surprised” (*rs* = 0.69, *p* < 0.0001), “disgusted” (*rs* = 0.49, *p* < 0.0001), “fearful” (*rs* = 0.39, *p* < 0.0001), “neutral” (*rs* = 0.25, *p* = 0.00037), “sad” (*rs* = 0.18, *p* = 0.0090, n.s. with Bonferroni correction), and “angry” (*rs* = 0.12, *p* = 0.094, n.s. with Bonferroni correction). The profiles of the two groups of the CNNs were similar to each other to varying degrees in the “happy,” “surprised,” “disgusted,” “fearful,” and “neutral” units, but differed substantially in the “sad” and “angry” units.

**Figure 8:**
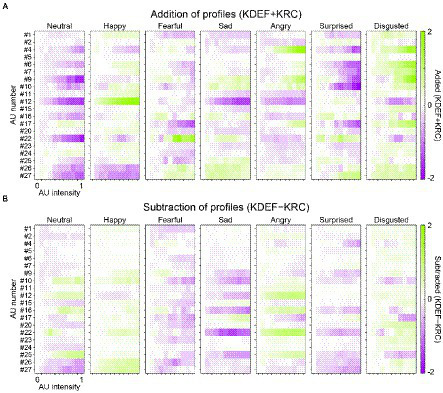
Similar and dissimilar tunings between the KDEF-trained and KRC-trained CNNs. For each of the seven computational units in the FC3 layer, the response profiles of the two groups of the CNNs are either added (KDEF + KRC) **(A)** or subtracted (KDEF − KRC) **(B)**. In A, positive and negative responses shared by the two groups are represented by darker green and purple colors, respectively. In B, green or purple indicates opposite tuning between the two groups. Green indicates positive responses in the KDEF-trained CNNs and negative responses in the KRC-trained CNNs. Purple indicates negative responses in the KDEF-trained CNNs and positive responses in the KRC-trained CNNs.

The overall similarity or dissimilarity of AU tunings between the KDEF-trained and KRC-trained CNNs can be more explicitly shown by plotting the addition or subtraction of the AU-tuning profiles of the same facial expression in the two groups of CNNs (Figure 8). We found some positive responses (green color in the panels of Figure 8A) in the added profiles, indicating shared tunings between the CNNs trained with the different databases. For instance, in agreement with Figures 5A,D, AU#12 (lip corner puller) had a strong positive effect on the added profile of the “happy” units. Another example of shared positive responses was AU#22 (lip funneler) in the profile of the “fearful” units and AU#4 (brow lowerer) in the profiles of the “angry” units. Additionally, in the profile of the “disgusted” units, several AUs, such as AU#4 and AU#9 (the latter of which indicates nose wrinkler), showed positive responses. We also found negative responses (purple colors in Figure 8A) shared by the two CNNs. For instance, AU#12 caused negative responses in the added profiles of the “neutral,” “sad,” “angry,” and “disgusted” units, in contrast to the strongly positive responses of the “happy” unit. Note that the added profile of the “neutral” unit was dominated by negative responses. This is reasonable, because the appearance of any facial movements implies that the face is not neutral but demonstrates some other facial expressions.

Subtraction of AU-tuning profiles of the same facial expression (KDEF − KRC) demonstrated opposite responses between the two CNNs (coded by either green or purple color in Figure 8B; green for larger responses in KDEF than in KRC, purple for larger responses in KRC than in KDEF). For instance, AU#22 (lip funneler) clearly elicited opposite effects between the KDEF-trained and KRC-trained CNNs in the profiles of the “sad” and “angry” units. The “sad” unit in the KDEF-trained CNN responded negatively to AU#22, while the “sad” unit in the KRC-trained CNN responded positively (compare “sad” panels in Figures 7A, B). For the “angry” unit, by contrast, the KDEF-trained and KRC-trained CNNs showed positive and negative responses, respectively, as already shown in Figures 6B,E.

### Relation of response profile similarities to confusion matrices

**Figure 9:**
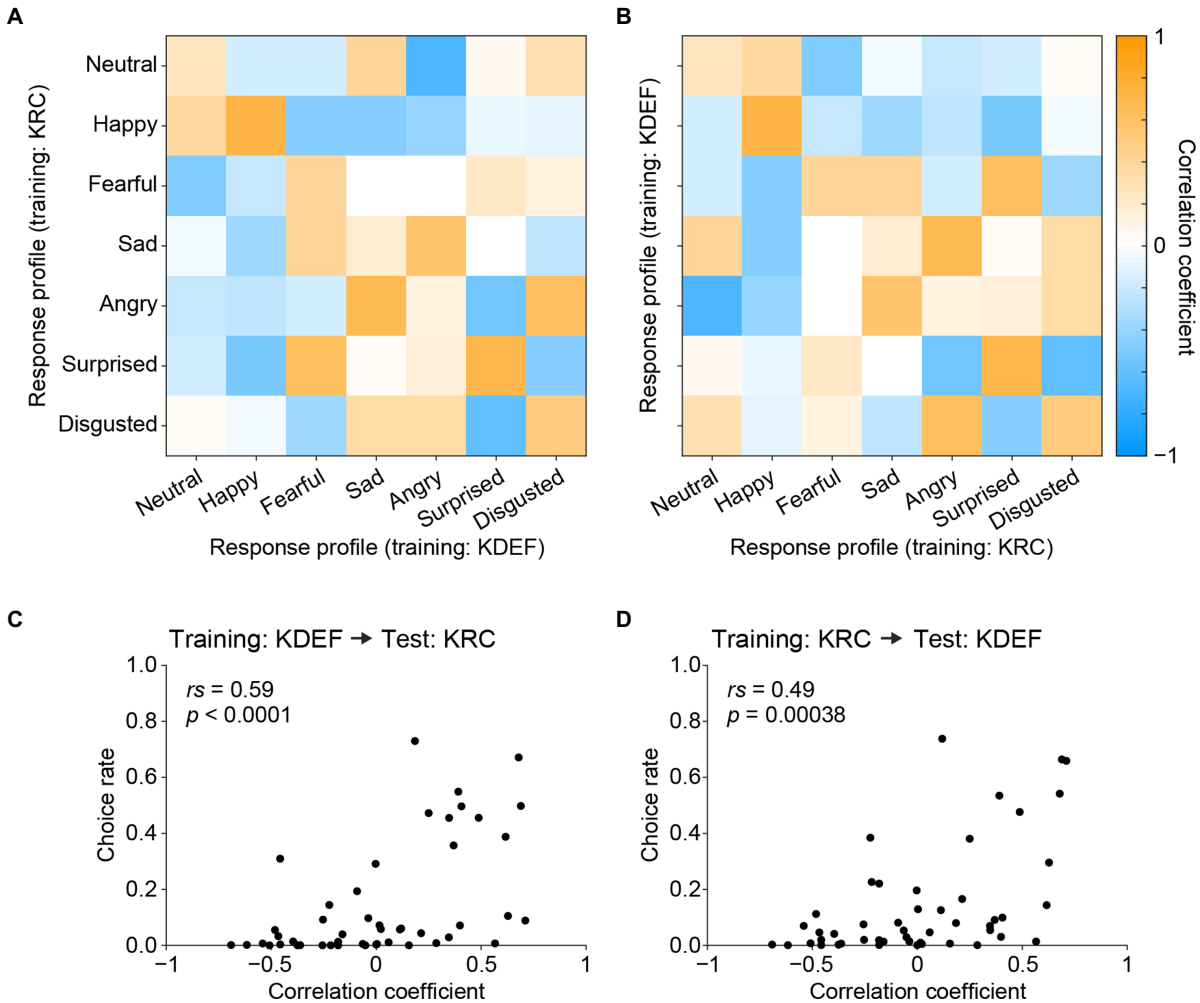
Correlation matrix of the response profiles of the KDEF-trained and KRC-trained CNNs and its relation to the confusion matrix in the database-swapped conditions. **(A,B)** Correlation matrix computed with the full profiles between the KDEF-trained and KRC-trained CNNs. Note that the horizontal axis and vertical axis are interchanged between **(A)** and **(B)**, while correlation values themselves are unchanged. **(C,D)** Correlation between the correlation matrix and confusion matrix in the database-swapped conditions. There are 49 (7 × 7) data points in the scatter plots, each data point representing the combination of two facial-expression units.

A crucial question is whether the similarities and dissimilarities of the AU-response profiles between the KDEF-trained and KRC-trained CNNs (Figures 7, 8) accounted for the confusion matrices in the database-swapped conditions (Figure 4). We computed the pairwise correlation coefficients between the two sets of seven profiles each of which was obtained from KDEF-trained and KRC-trained CNNs, thus resulting in a 7 × 7 matrix (Figure 9). Note that the vertical axis of the correlation matrix (corresponding to the “true” label in the confusion matrix) was set to the profiles of the KRC-trained CNNs in Figure 9A, and to those of the KDEF-trained CNNs in Figure 9B. As already described above in Figure 7, the correlations of the pairs of the same facial expression, which lie along the principal diagonal, showed a variety of coefficient values, some of which were fairly high. Several combinations of facial expressions, such as the pair of the “sad” profile in the KDEF-trained CNNs and the “angry” profile in the KRC-trained CNNs, demonstrated higher correlation coefficients (shown in orange in Figures 9A,B) than other combinations (shown in blue and white). If the selectivity to the AUs underlies the classification performance of the CNNs, one might expect that pairs with higher correlation in terms of the AU selectivity would show higher choice rates in the confusion matrix. This was indeed the case. Higher correlation coefficients were associated with higher choice rates in both database-swapped conditions (Figures 9C,D; KDEF to KRC, *rs* = 0.59, *p* < 0.0001; KRC to KDEF, *rs* = 0.49, *p* = 0.00038). These results suggest that the classification of the facial expressions by the CNNs was at least partly mediated by the selectivity to the AUs.

### Dimension reduction by principal component analysis

**Figure 10:**
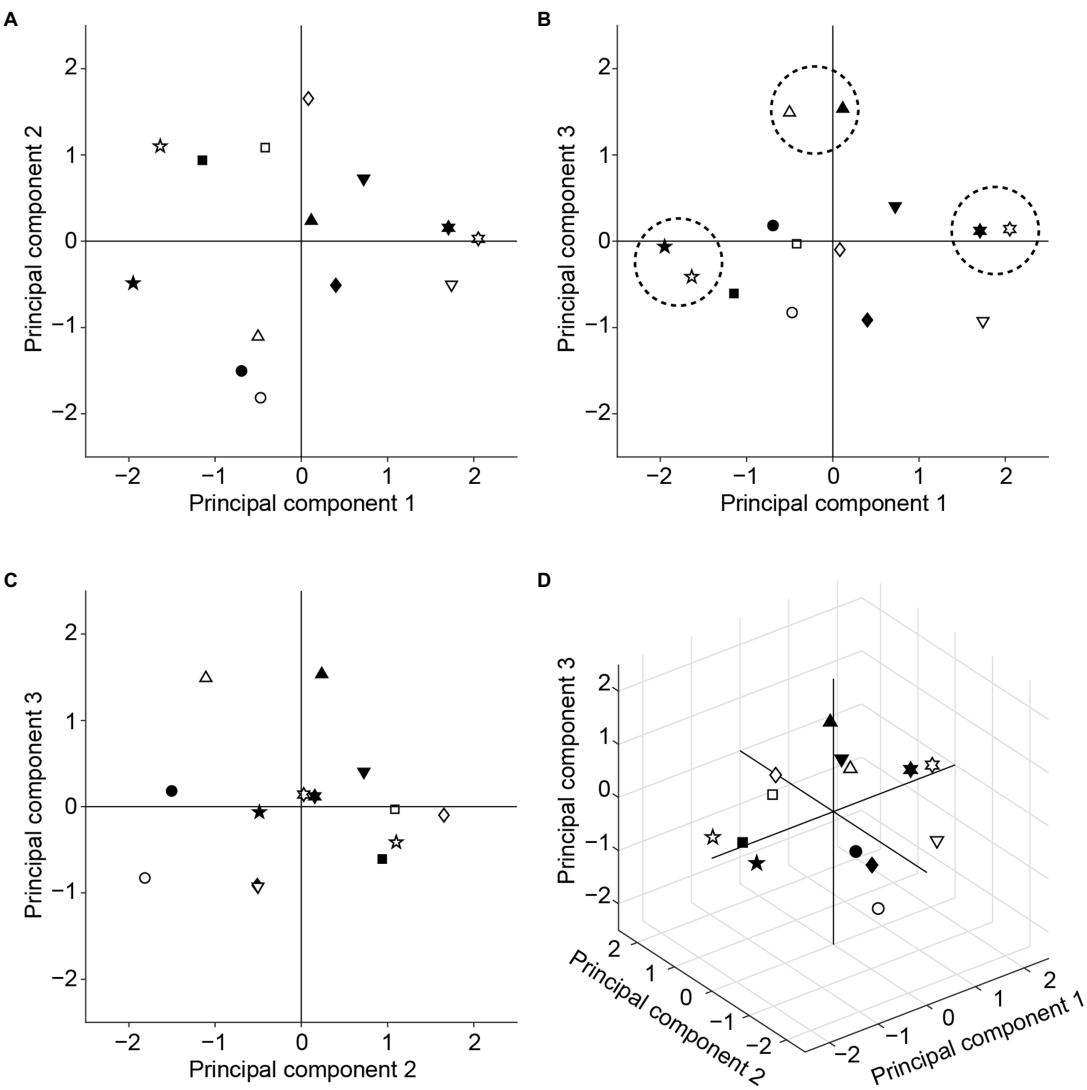
Principal component analysis of the AU selectivity of the computational units in the FC3 layer. Distribution of the response profiles of the facial expressions in two-dimensional spaces spanned by principal components 1 and 2 **(A)**, principal components 1 and 3 **(B)**, and principal components 2 and 3 **(C)**. The distribution in the three-dimensional space is shown in **(D)**. Symbols with different shapes denote different facial expressions. Circles: neutral; upward-pointing triangles: happy; squares: fearful; diamonds: sad; downward-pointing triangles: angry; pentagons: surprised; hexagons: disgusted. Filled and open symbols indicate data from the KDEF-trained and KRC-trained CNNs, respectively. In **(B)**, the closely located pairs of expressions between the two databases (neutral, disgusted, surprised) are encircled by dashed lines.

Finally, we performed principal component analysis to visualize how selectivity of the seven facial-expression units to the AUs differed from each other. Figure 10 shows the distribution of the facial expressions in the two- or three-dimensional spaces spanned by the combinational pairs of the first three principal components (A–C) or by all of them (D). The differently shaped symbols indicate different facial expressions. Filled and open symbols indicate the data for the KDEF-trained and KRC-trained CNNs, respectively. Happy (upward-pointing triangles), surprised (pentagons), and disgusted (hexagons) faces were clustered in the vicinity between the KDEF and KRC (see dashed circles in Figure 10B). The other expressions were located at greater distances between the two databases. These figures may serve as an intuitive summary of the relationship between the facial expressions defined by AU selectivity.

## Discussion

In this study, we performed CNN-based image analysis to compare the features of facial expressions in different cultures. We trained CNNs to classify images of facial expressions in two image databases, one developed in Sweden and the other in Japan (KDEF and KRC, respectively). Sweden and Japan are far apart in distance and belong to European and Asian countries, respectively. Because differences in mental representations for facial expressions between Europeans and Asians have been suggested (Jack et al., 2012), we assumed that cultural differences were reflected in these two databases. After training with one of the two databases, the CNNs were able to classify facial expressions in the same database with high accuracy. When the database was swapped between the training and test datasets, however, the performance of the CNN was degraded, with frequent confusion between specific pairs of facial expressions. Classification performance was only partially transferable between the databases. These results suggest that the CNN trained with a different dataset exploited different facial features to classify the facial expressions. Analysis of the selectivity of the FC3 computational units to the AUs of facial movements revealed a variety of tunings to a subset of AUs (each either positive or negative). These tunings depended on the databases used for the training as well as on the facial expressions to be classified. The similarity or dissimilarity of these tunings across the databases and facial expressions was correlated with the confusion matrix of the CNN classifications in the database-swapped conditions. Taken together we show that the visual features of characterizing facial expressions differ between KDEF and KRC (hence, between Sweden and Japan), and the differences in associating specific AUs to particular expressions partially define this difference. These findings support the interpretation that our results reflect culture-specific differences in expressing emotions rather than differences of databases as such. In a more general context, the results demonstrate the usefulness of the AUs in characterizing facial features represented in the CNNs. With this justification, we summarized the representations of the facial expressions in the CNNs by applying principal component analysis to the AU selectivity. The distribution in this low-dimensional space may serve as an intuitive summary for systematic comparisons of different facial expressions in different databases.

### Comparison with classification data in humans

The CNN used in this study was very powerful, and its performance in classifying facial expressions was superior to that of humans. The grand averages of the correct rates across the facial expressions were 0.76 and 0.75 in the KDEF-trained and KRC-trained CNNs, respectively, and were higher than those in psychological validations of human observers (KDEF, 0.72, Goeleven et al., 2008; KRC, 0.62, Ueda et al., 2019). The CNNs effectively learned the visual features important for classification of facial expressions in the KDEF or KRC databases.

The correct rates depended on the facial expressions in the database-matched conditions (Figures 3C,D). As in the CNNs, classification accuracy in humans also varies across facial expressions. For the KDEF database, the mean correct rates across observers in a classification task were ranked in descending order as happy, angry, surprised, sad, disgusted, neutral, and fearful faces (Goeleven et al., 2008). This order is consistent with our data in that the highest and lowest correct rates were observed for happy and fearful faces, respectively (rank order: happy, neutral, surprised, disgusted, angry, sad, and fearful). For the KRC database, a classification experiment in human participants demonstrated that the rank order was neutral, surprised, happy, sad, disgusted, angry, and fearful faces (Ueda et al., 2019). The top three facial expressions were the same as in our data (rank order: happy, surprised, neutral, fearful, disgusted, sad, and angry). Patterns of the correct rates were partially matched between the CNNs and human observers.

### Tuning to AUs

The classification ability of the CNNs was not fully generalized between the databases: the CNNs trained with one of the two databases made frequent mistakes in classifying the facial expressions when test images were drawn from the other database (Figure 4). These results suggest that facial features associated with each facial expression may differ between the two databases. To address this issue, we characterized a face by FACS (Ekman and Friesen, 1976; Ekman et al., 2002), which codes facial movement by a set of AU intensities. We took this approach for multiple reasons. First, an AU-based representation has only a few tens of components (i.e., AUs) and can efficiently generate facial expressions because of the close relationship of AUs with the anatomy of the face. These advantages were highlighted by the fact that many psychological studies have adopted this framework (see Barrett et al., 2019, for a review). Second, an electrophysiological study in non-human primates suggested that some neurons of a face selective region (middle face patch) in the visual temporal cortex encode AU-like components of facial movement (Freiwald et al., 2009). Such AU selectivity may constitute precursors for the facial-expression selectivity exhibited by some neurons in downstream areas (Hasselmo et al., 1989; Nakamura et al., 1994; Sugase et al., 1999; Gothard et al., 2007; Hoffman et al., 2007; Inagaki et al., 2022). Third, in CNNs, the performance of classifying facial expressions can be improved by pruning feature maps that are not selective to AUs (Zhou and Shi, 2017). Indeed, in our data, the tunings to the AUs were related to the prediction of the facial expressions by the CNNs in the database-swapped conditions (Figure 9).

FC 3 units exhibited both positive and negative responses to higher AU intensities that are accompanied by the clear expression of corresponding facial movements in an image. A positive response (i.e., stronger than that to the null image) to a specific AU in a specific facial-expression unit indicates that the existence of the corresponding facial movement promotes classification of the input face as this facial expression. Note that positive responses captured only the linear effects of AUs and not the nonlinear interactions among AUs, because AUs were individually manipulated to obtain each AU tuning. The nonlinear interactions would happen to a combination of multiple AUs, each localized at different positions in a face (e.g., eye region vs. mouth region). Although the interactions were potentially informative factors as well, our approach clarified the representation of the AUs in the FC3 units as a first order approximation. Some of the positive responses were consistent with the original proposal of the relation between specific AUs and expressions (Ekman and Friesen, 1976; Ekman et al., 2002). For instance, both the KDEF-trained CNNs and KRC-trained CNNs showed positive responses to AU#12 (lip corner puller) in the “happy” unit, as AU#12 is suggested by the FACS guide to be indicative of a happy facial expression (Figure 8A, panel “Happy”). Both groups of CNNs also responded positively to AU#4 (brow lowerer) in the “angry” unit, which is associated with an angry facial expression according to the guide (Figure 8A, panel “Angry”). Among the positive responses in the “disgusted” unit (Figure 8A, panel “Disgusted”), only that to AU#9 (nose wrinkler) was supported by the guide. However, other positive responses in this unit, such as to AU#4, AU#6 (cheek raiser), and AU#7 (lid tightener), were supported by another study (Cordaro et al., 2018) in which facial movements elicited by emotional stories were analyzed by human raters. Overall, the pattern of the positive responses was partly matched to the AU-based characterization of facial expressions (Ekman et al., 2002; see Barrett et al., 2019, for a review).

A negative response (i.e., weaker than that to the null image) indicates that the existence of the corresponding facial movement inhibits the input face from being classified as this facial expression. Negative responses might thus contribute to correctly rejecting irrelevant facial expressions, although it is unclear exactly how their patterns are related to the original FACS guide. This uncertainty does not mean that negative responses are useless for analyzing image databases. Rather, the negative response patterns found in the present study might facilitate the characterization of facial expressions according to AUs. This is because in a psychological examination, it is difficult and time-consuming, if not impossible, to confirm whether a specific AU induces rejection of irrelevant facial expressions. Our analysis might shed light on a hitherto unexplored aspect of the relationships between AUs and facial expressions.

The tunings to the AUs markedly differed between the KDEF-trained and KRC-trained CNNs (Figure 8B). Because one of our aims was to build an image analysis framework that can be used to compare many image databases, we need a compact summary of the AU tunings obtained from different databases. For this purpose, we applied principal component analysis (Figure 10). Distributions of facial expressions in the low-dimensional space deduced from principal component analysis suggest that neutral, disgusted, and surprised faces are similar between the two databases compared to the other facial expressions (Figure 10). Application of this analysis to various databases may give an intuitive overview of the similarities and dissimilarities of the facial expressions based on the AUs across different countries or cultures.

A potential concern of our approach is a sampling bias of the face images used for the analysis. We selected images of 60 individuals each from the KDEF and KRC databases to compare the two databases using a fixed number of images in the main analysis. A small sample size may fail to capture the large variation in the AU tunings among individuals of the target population. To address this issue we increased the number of face images by using all available data (70 individuals in KDEF, 74 individuals in KRC) and compared the AU tunings of the FC3 units with those of the main data (obtained from 60 individuals). For the KDEF database, the correlation coefficients between the response profiles obtained from the two conditions (60 individuals vs. 70 individuals) ranged from 0.967 to 0.998 for the seven facial expressions. For the KRC database, the correlation coefficients between the two conditions (60 individuals vs. 74 individuals) were from 0.985 to 0.997. The response profiles were quite similar between different sample sizes, suggesting that the main data based on the images of 60 individuals captured the major variations in these databases.

Another concern is a possible difference in the intensity of facial expressions. If a database consists of face images only faintly expressing emotions, one might fail to assess the features of facial expressions. The previous validation studies showed that the mean score of the intensity of the six basic emotions rated by human observers ranged from 5.28 to 6.23 (tested with 9 point Likert scale) in the KDEF database (Goeleven et al., 2008) and from 4.19 to 5.32 (tested with 7 point Likert scale) in the KRC database (Ueda et al., 2019). The intensity scores were moderate in both databases on average. These findings suggested that the face images in these two databases expressed the basic emotions with a similar intensity level, and thus they were suitable for analyzing the visual features of the facial expressions.

### Image analysis of facial expressions

A previous study on human participants used a biologically plausible neural network to infer mechanisms underlying high in-group recognition accuracy of facial expressions (i.e., Japanese participants show higher correct rates for expressions by Japanese, while American participants show higher correct rates for expressions by Americans; Dailey et al., 2010). They trained their neural network with dataset mixtures consisting of different proportions of American and Japanese faces, and reproduced this in-group advantage of facial expression recognition. In agreement with their finding, the performance of our CNNs was higher in the database-matched conditions than in the database-swapped conditions. We further showed that the tunings of the output units to AUs differed between the KDEF-trained and KRC-trained CNNs. The specialization to different sets of AUs may underlie the higher performance in the data-matched conditions, suggesting that people in different cultures develop specific sensitivity to AUs.

The outputs of the FC3 layer of our CNNs were selective to AUs. A previous study showed that some portions of the outputs of the CONV5 layer (after max pooling), which is located upstream of the FC layers, are already selective to AUs (Zhou and Shi, 2017). As in our CNNs, their CONV5 layer is pre-trained with the ImageNet database (Russakovsky et al., 2015) and not trained with a face database. Therefore, the selectivity of the outputs of CONV5 layer to AUs must have been obtained through learning with a variety of object and scene images in the ImageNet database without dense exposure to images of facial expressions. The AU-selectivity in the pre-trained CONV5 layer may contribute to classification of facial expressions through the FC layers which learn in the training with face databases how to combine the AU-selective outputs from the CONV5 layer. This transferability of the learning by CONV layers from pre-training to facial-expression discrimination training is beneficial because training of the entire CNN including both CONV and FC layers with a face database is time-consuming and requires a large number of images of facial expressions.

Recently, software programs of automated scoring have developed to evaluate AUs of faces (den Uyl and van Kuilenburg, 2005; Littlewort et al., 2011; Olderbak et al., 2014; Girard et al., 2015). These automatic analyses are applied to images of faces to determine whether and how each AU appears in them. In the present study we also developed a framework of automatic analysis of AUs, which was, however, not directly applied to images of faces. Instead we tested the AU selectivity of the outputs of the CNNs trained with images of faces. Because the CNNs were optimized to the facial-expression classification, their output selectivity reflected diagnostic information of facial features useful for discriminating different facial expressions. The response profiles for the AUs shown in Figure 7, therefore, characterized how effectively each AU differentiates the corresponding facial expression from the others in a specific database used for the training. Our method might shape the profiles more compact compared with those obtained from other analyses working on images themselves.

In conclusion, we developed a novel method of CNN-based image analysis to determine visual features characterizing facial expressions. We applied this analysis to facial image databases developed in different countries. The technical merits of this approach are that emotion labels annotated for face photographs provide clues for understanding culture-specific relations between facial movement patterns and facial expressions, and that image analysis can be performed without any experimenter bias. We confirmed the validity of our method by demonstrating that the visual features of characterizing facial expressions differ between two databases developed in Sweden and Japan. Our framework of image analysis can be easily extended to new databases when they become available, and will facilitate systematic comparisons of visual features characterizing facial expressions across different cultures.

## Data availability statement

The raw data supporting the conclusions of this article will be made available by the authors, without undue reservation.

## Data availability statement

The raw data supporting the conclusions of this article will be made available by the authors, without undue reservation.

## Ethics statement

Written informed consent was obtained from the individual(s) for the publication of any identifiable images or data included in this article.

## Funding

This research was supported by Grant-in-Aid for Scientific Research on Innovative Areas “Construction of the Face-Body Studies in Transcultural Conditions” (JP18H04197 and JP20H04578) to MI, and by Grant-in-Aid from Japanese Ministry of Education, Culture, Sports, Science, and Technology (JP21H02596) to IF.

## Conflict of interest

The authors declare that the research was conducted in the absence of any commercial or financial relationships that could be construed as a potential conflict of interest.

## Publisher’s note

All claims expressed in this article are solely those of the authors and do not necessarily represent those of their affiliated organizations, or those of the publisher, the editors and the reviewers. Any product that may be evaluated in this article, or claim that may be made by its manufacturer, is not guaranteed or endorsed by the publisher.

## Footnotes

^1^https://opencv.org/ (accessed October 7, 2022).
